# From Clone Selection to 80L Bioreactor Production: Better, Faster, Leaner

**DOI:** 10.1186/1753-6561-9-S9-P34

**Published:** 2015-12-14

**Authors:** Marie-Françoise Clincke, Frédéric Delouvroy, Guillaume L Révérend, Jimmy Stofferis, Matthew Hinchliffe, Laetitia Malphettes

**Affiliations:** 1 Upstream Process Sciences, BioTech Sciences, UCB Pharma S.A., Braine L'Alleud, Belgium; 2 Biology Research Discovery, Protein Expression & Purification, UCB Celltech, Slough, UK

## Background

Bio-pharmaceutical industries face an increasing demand to accelerate process development and reduce costs. Indeed as technologies mature, pressure on cost and timelines is greater for delivering scalable and robust processes. Here we present a novel method in order to speed up and interconnect clone selection with cell culture process development and scale up to 80L scale.

## Materials and methods

Ambr™ 48 and 80L stirred tank bioreactor were run for 14 days in a fed-batch mode in a chemically defined medium. A CHO cell line expressing a recombinant monoclonal antibody (MAb) was used. Feed was added daily from day 3 onwards. If required, antifoam C was added to the bioreactor. DO, pH, and temperature were controlled at setpoint. Viable cell concentration (VCC), cell viability, and average cell diameter were measured using a ViCell cell counter. Osmolality was obtained using an Osmometer (Advanced Instruments). On harvest day, MAb concentration of the supernatant samples was quantified using Protein A high performance liquid chromatography. For the minibioreactors run, triplicates were pooled on harvest day for product quality attributes analysis. For both scales cell culture fluid samples were centrifuged and filtered to remove cell debris. The monoclonal antibody was purified by ÄKTAXpress (GE Healthcare) Protein-A purification. The neutralized eluate was used for product quality analysis.

## Results

High throughput technologies enable us to interconnect clone and process development thus reducing the risks associated with early-stage process development while reducing timelines and enabling us to achieve higher process robustness in a leaner manner. In one ambr™48 run different feeding strategies were assessed on 4 pre-selected lead clones for selecting the final lead clone, the fed-batch feeding strategy and additional data with feed compositions all at once. The selected condition (clone, type of feed and feeding strategy) was based on the maximization of the MAb titer and target High Molecular Weight Species (HMWS) level and Acidic Peak Group (APG) level (data not shown).

**Figure 1:**
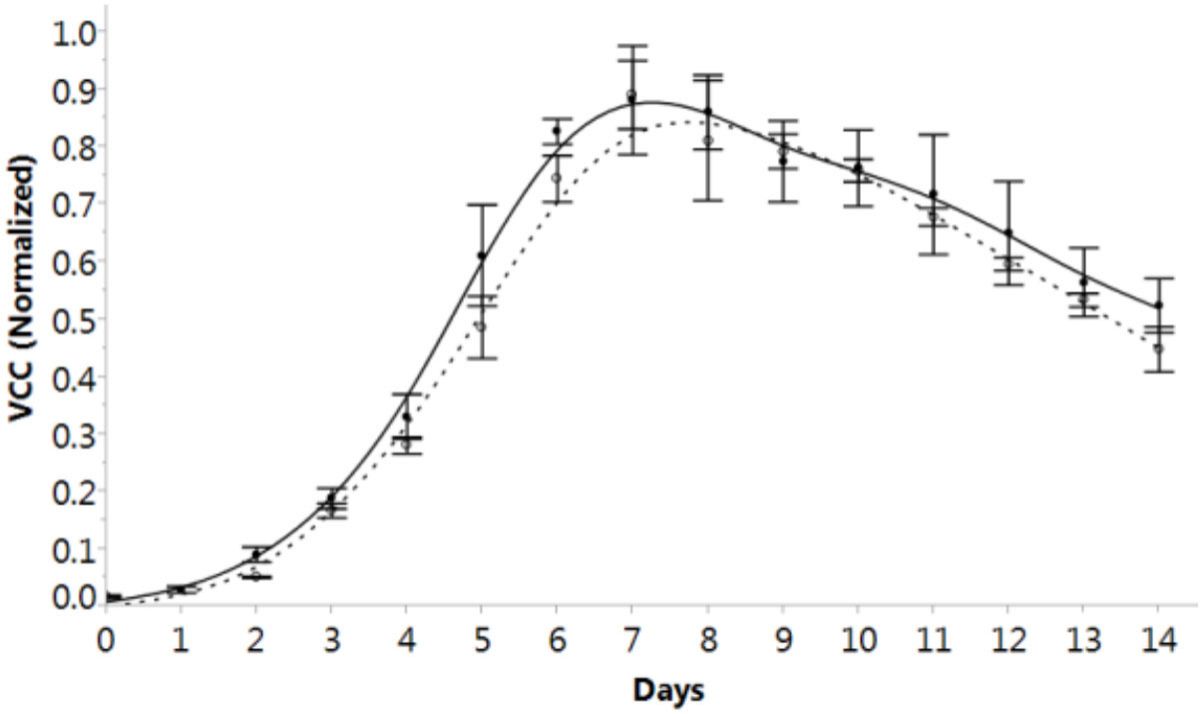
**Viable cell concentration comparison between ambr™ and 80L scales (solid black line: 80L scale, dotted black line: ambr™scale)**.

Scale up from ambr™ 48 to 80L stirred tank bioreactor was performed with the selected clone, feed and feeding strategy. Similar cell growth profiles were obtained at ambr™ scale and 80L scale for the selected condition (Figure 1).

Monoclonal Antibody titers and percentages of Acidic Peak Group obtained at harvest day were comparable between scales (Table 1). Process robustness was shown by performing a number of 4 batches at 80L scale.

**Table 1:** MAbtiters and APG level obtained with the selected clone, feed and feeding strategy on harvest day at ambr™ and 80L scales

	MAb titer (Normalized)	APG (Normalized)
ambr™ (n = 3)	0.9	0.7
80L (n = 4)	0.8	0.7

## Conclusions

Selection of the lead clone together with the optimal feeding strategy was performed in one single ambr™ run and 16 conditions were tested in triplicate at the time of clone selection, compared to conventional approaches relying on 1L-10L stirred tank bioreactor experiments run on a single lead clone. Scale up was achieved successfully thus enabling early material production for downstream, formulation and analytical development. In conclusion, miniaturized bioreactors enabled us to interconnect clone and process design space selection and enable immediate 80L production.

